# Design, synthesis, and characterization of novel eco-friendly chitosan-AgIO_3_ bionanocomposite and study its antibacterial activity

**DOI:** 10.1038/s41598-022-14501-6

**Published:** 2022-06-21

**Authors:** Mohammad Ali Ahghari, Mohammad Reza Ahghari, Maryam Kamalzare, Ali Maleki

**Affiliations:** Catalysts and Organic Synthesis Research Laboratory, Department of Chemistry, Iran University of Science and Technology, Tehran, 16846-13114 Iran

**Keywords:** Biochemistry, Chemical biology, Chemistry, Materials science, Nanoscience and technology

## Abstract

This work reports a facile and green approach to preparing AgIO_3_ nanoparticles decorated with chitosan (chitosan-AgIO_3_). The bionanocomposite was fully characterized by Fourier transform infrared (FTIR), scanning electron microscopy (SEM) images, energy-dispersive X-ray spectroscopy (EDX), and X-ray diffraction analysis (XRD). The antibacterial effect of chitosan-AgIO_3_ bionanocomposite was investigated for *Pseudomonas aeruginosa, Klebsiella pneumoniae*, *Staphylococcus saprophyticus*, *Escherichia coli,* and *Staphylococcus aureus* as pathogen microorganisms via the plate count method, disk diffusion method, and optical density (OD) measurements. The antibacterial performance of the bionanocomposite was compared with two commercial drugs (penicillin and silver sulfadiazine) and in some cases, the synthesized bionanocomposite has a better effect in the eradication of bacteria. The bionanocomposite represented great antibacterial properties. Flow cytometry was performed to investigate the mechanism of bionanocomposite as an antibacterial agent. Reactive oxygen species (ROS) production was responsible for the bactericidal mechanisms. These results demonstrate that the chitosan-AgIO_3_ bionanocomposite, as a kind of antibacterial material, got potential for application in a broad range of biomedical applications and water purification. The design and synthesis of green and biodegradable antibacterial materials with simple processes and by using readily available materials cause the final product to be economically affordable and could be scaled in different industries.

## Introduction

**Figure 1:**
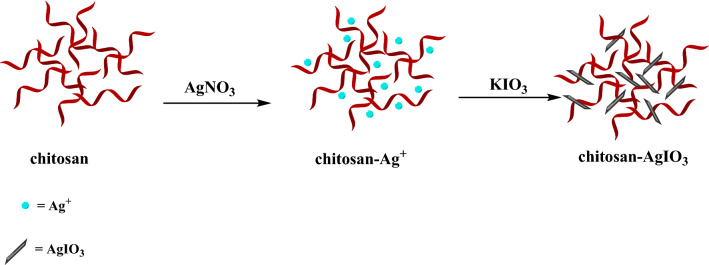
The synthesis process of chitosan-AgIO_3_ bionanocomposite.

Regarding the spread of infectious disease attributable to pathogenic bacteria and the rise of antibiotic resistance, demand for the design and synthesis of unique antibacterial agents is increased^1^. *Pseudomonas aeruginosa* is a pernicious pathogen as a gram-negative aerobic bacillus isolated from soil, water, plants, and animals, including humans. *Pseudomonas aeruginosa* is also known to change its phenotype and attune to the environment and are multi-drug resistant bacterial species, affecting compromised immune system patients^2,3^. *Klebsiella pneumoniae* is a rod-shaped, gram-negative pathogen extensively found in the mouth, skin, intestines, in-hospital settings, and medical devices. The opportunistic pathogen *Klebsiella pneumoniae* mostly influences those with compromised immune systems or is weakened by other infections. Considering that *Klebsiella pneumoniae* has become increasingly resistant to antibiotics, successful eradication of this bacterium is very important^4,5^. *Staphylococcus saprophyticus* is related to uncomplicated urinary tract infection in humans^6^. *Escherichia coli* (gram-negative) and *Staphylococcus aureus* (gram-positive) cause diarrhea diseases in humans after infected water. A safe drinking water supply is a critical and essential aspect of human health^7^. Silver sulfadiazine (AgSD) has been used for the treatment of second-order burns since the early 1970s. It represents a more effective antibacterial dressing with improved stimulation of wound regeneration^8,9^. Penicillin is a β-lactam antibiotic that is useful against a wide range of bacteria. Penicillins are the drug of choice for upper and lower respiratory infections (*Streptococcus pyogenes*), meningococcal disease (*Neisseria meningitides*), syphilis (*Treponema pallidum*), and anaerobic infections^10^. The field of nanotechnology has been introduced as a high potential possibility for producing novel nanoscale materials which represent high surface to volume area and particular physical and chemical properties with wide applications^11^. Amongst them, a DNA nanostructure electrochemical biosensor was designed and synthesized for monitoring cyanazine herbicide in water and food samples^12^. Nitrogen and sulfur co-doped carbon dots were effectively synthesized from waste orange peels via a cost-natural and easy synthesis process as a nano-booster with high oxygen-reduction reaction activity^13^. Besides other oxygen-reduction reactions electrocatalyst in neutral media was designed and synthesized as an effective alternative to expensive metal-based nanocatalysts^14^. A novel carbon paste electrode modified with ZIF-8/g-C_3_N_4_/Co nanocomposite and 1-methyl-3-butylimidazolium bromide as an ionic liquid was utilized as an extremely sensitive electrochemical sensor for the detection of synthetic azo dyes^15^. A guanine-based DNA biosensor was designed and fabricated in a simple way for monitoring anticancer drugs during chemotherapy treatments^16^. Nanoparticles as an antibacterial agent with a large surface area to volume ratio that can provide better contact with bacterial cells. Considering that nanoparticles tend to aggregate, which could reduce the antibacterial property, utilizing support for nanoparticles will be very practical and helpful in producing a nanocomposite with high antibacterial efficacy^17,18^. Green nanotechnology supplies many superiorities in terms of process development, manufacturing, and product design. The synthesis of nanocomposite in the direction of green chemistry has many preponderances, including simple and mild reaction conditions that cause the process to be scaled up, economically affordable, and eco-friendly^19^. Bionanocomposite could be used in several fields, such as antibacterial agents^20^, bionanocatalysts^21^, photocatalytic activity^22^, adsorbents for heavy metals^23^, and drug delivery^24^. With a view to green chemistry, extending and utilizing biodegradable polymers is considered the most thorough method for designing bionanocomposites as an antibacterial agent. Polymers derived from natural resources, including starch, cellulose, chitin, chitosan, and lignin have emerged as promising candidates for synthesizing bionanocomposite with particular applications^25–28^. As a natural polymer derived from the marine environment, chitosan is an amino polysaccharide obtained by the deacetylation of chitin (poly-N-acetyl-D-glucosamine). Chitin is the second most abundant natural polymer after cellulose. Chitosan is the most beneficial derivative of chitin with numerous amine and hydroxyl groups in its structure, which enables the synthesis of biocomposite with various applications. Chitosan represented special features, including biodegradability, biocompatibility, nontoxicity, inexpensiveness, and availability^29,30^. Among the different antibacterial nanomaterials, silver nanoparticles and their compounds have substantial antimicrobial capabilities. Silver nanoparticles (AgNPs) attracted the attention of many researchers due to their antimicrobial activity against a wide variety of drug-resistant microorganisms^31^. Gold/silver–tellurium nanostructures (Au/Ag − Te NSs) has very good antimicrobial activity against different microorganism due to the generated ROS which destroys the bacteria membrane^32^. Silver nanoparticle anchored graphene oxide (GO-Ag) has shown good antibacterial activity^33^, Vancomycin capped with silver nanoparticles as an antibacterial agent^34^, Nanowires of silver–polyaniline bionanocomposite as an antibacterial agent^35^, cellulose/γ-Fe_2_O_3_/Ag bionanocomposite as an antibacterial agent^36^, are some of the examples of silver nanocomposite with antibacterial activity. AgIO_3_ is an insoluble white crystal with an orthorhombic structure representing good photocatalytic activity for the decomposition of organic pollutants in the UV region because of its wide bandgap and high separation rate of photoexcited charge carriers^37–39^. Silver iodate nanoparticles are highly insoluble (AgIO_3_ K_sp_ = 3.1 × 10^–8^) and could be used in water treatment as well as medical applications^40^. In connection with our previous research on the antibacterial activity of nanocomposite^41–43^, in this study, we present the preparation and identification of chitosan-AgIO_3_ bionanocomposite as an antibacterial agent (Fig. 1). To the best of our knowledge, this is the first report on the antibacterial properties of bionanocomposite based on chitosan and silver iodate against several gram-negative and gram-positive bacteria. Chitosan-AgIO_3_ bionanocomposite was introduced as a unique, cost-effective with high antibacterial activity. This paper opens a new approach in an antibacterial field involving economically and environmentally efficient nanoscale composite based on natural polymer. Moreover, the synthesized bionanocomposite could be employed in broad usages and could be scale-up due to its novel and special properties. Fabricating nanomaterials with available and green resources causes biodegradability and biocompatibility and reduces the cost of the synthesized products. Simple equipment and procedure and inexpensive and readily available materials without the use of any surfactants, external templates, and toxic solvents for synthesizing the bionanocomposite are of great importance. In order to acknowledge and emphasize on antibacterial activity of the chitosan-AgIO_3_ bionanocomposite, the antibacterial performance of the bionanocomposite was compared with two commercial antibiotics against five human pathogenic bacteria. Chitosan-AgIO_3_ bionanocomposite has desirable antibacterial activity compared to penicillin against five different bacteria and in case of *Pseudomonas aeruginosa* and *E.coli* even has better performance. Chitosan silver iodate bionanocomposite was checked with silver sulfadiazine against five bacteria and it was determined that bionanocomposite has a better effect against *Pseudomonas aeruginosa* and also it has equal efficacy in *Klebsiella pneumoniae.*

## Experimental

### General

The solvents, chemicals, and reagents were acquired from various commercial companies such as Merck, Sigma-Aldrich, and Fluka and were used as received. Penicillin G 800 000 U was provided from Jaber Ebne Hayyan Pharmaceutical Company (Iran). FT-IR spectra were recorded using Shimadzu IR-470 spectrometer in the transmission mode using KBr pellets of the sample. Elemental analysis of prepared samples was performed by EDX analysis recorded on Numerix DXP-X10P. The morphology and structure of the bionanocomposite were studied by SEM, VEGA2 TESCAN instrument. The XRD patterns of the solid powders were developed using a JEOL JDX-8030 (30 kV, 20 mA). The optical properties of samples were measured with a Shimadzu UV–visible Mini 1240 spectrophotometer. An autoclave (Reyhan Teb, 2KW-220v) was used to sterilize the glassware. An incubator (Sh, Noor Sanat Ferdos) was used for cell cultivation. The flow cytometry data were acquired using a BD FACSCalibur (BD Biosciences, San Jose, CA, USA). 2′-7′-Dichlorodihydrofluorescein diacetate (DCFH-DA) was used as a detection probe to assess ROS generation for the study of the antibacterial mechanism of bionanocomposite.

### Synthesis of chitosan-AgIO_3_ bionanocomposite

For the synthesis of chitosan-AgIO_3_ bionanocomposite, 0.16 g AgNO_3_ was dissolved in 10 ml of deionized water, then added to the 10 ml of 0.5% (m/v) chitosan solution and stirred for 30 min in a dark condition to adsorb Ag^+^ ions. In the following 0.21 g, KIO_3_ was dissolved in 10 ml deionized water, gradually added to the above solution, and stirred for 3 h. A milky white solid was obtained and washed with deionized water and ethanol and dried at 70 °C for 12 h.

### Procedure for antibacterial studies with chitosan-AgIO_3_ bionanocomposite

For the study, the antimicrobial performance of the synthesized bionanocomposite, standard agar diffusion test, colony count method, and antibacterial activity screening (OD study), was performed against various bacteria, including *Pseudomonas aeruginosa* (ATCC 27853*), Klebsiella pneumoniae* (ATCC 700603)*, Staphylococcus saprophyticus* (ATCC 1440)*, Escherichia coli* (ATCC 9637)*, Staphylococcus aureus* (ATCC 12600). Besides, the antibacterial performance of chitosan silver iodate was compared with penicillin and silver sulfadiazine by utilizing a standard agar diffusion test. All instruments have been sterilized at 121 °C for 10 min in an autoclave before any process. Further, 0.5 McFarland turbidity standard to Nutrient Broth media was applied for antibacterial tests.

#### Antibacterial activity screening (ZOI study)

Muller-Hinton agar was utilized as a base medium and a solid growth medium plus nutrients microorganisms; 20 ml of Muller-Hinton agar was added to the plate (8 cm) and 50 ml was added to the other plate (15 cm) in the sterile condition. Five different tube test which was filled with 10 ml of sterile physiological serum and each of the five microorganisms was adjusted with 0.5 McFarland turbidity, then with utilizing a sterile glass hockey stick, the culture (cell concentration was adjusted to 10^7^ cells/mL) was dispersed around the surface of the plate. In the following the 0.01 g of chitosan-AgIO_3_ was added to the Muller-Hinton agar culture medium containing bacteria. Petri dishes containing bacteria and bionanocomposite were incubated for 24 h at 37 °C. To evaluate the antibacterial effects of the bionanocomposite against these five types of bacteria, and compared it with commercial drugs plates consisting of penicillin and silver sulfadiazine (with different concentrations) were studied in the separate plate which was prepared as mentioned above. The growth inhibition zones of chitosan-AgIO_3_ bionanocomposite against *Pseudomonas aeruginosa, Klebsiella pneumoniae, Staphylococcus saprophyticus, Escherichia coli,* and *Staphylococcus aureus* were measured.

#### Colony count method

One of the most practical and useful methods for studying the effect of bionanocomposite as an antibacterial agent against bacteria is the colony count method. *Escherichia coli* and *Staphylococcus aureus* were grown for 24 h in Muller-Hinton agar and were used for the colony count method. Then two tube tests containing these bacteria and 10 ml sterile physiological serum were adjusted with 0.5 McFarland turbidity standard. Each of these tube tests was diluted three times with sterile physiological serum. Then 0.1 mℓ of DMSO was added to four flasks of Nutrient Broth culture media. The obtained opacity solution was then divided into two portions for each bacterium and placed in two different flasks. Bionanocomposite (0.01 g) was added to one of the solutions of each bacterium in a flask, and the remaining flask has no bionanocomposite. Therefore two flasks have bacterium and bionanocomposite and two of them have no bionanocomposite, the flasks have just bacterium introduced as proof for comparison with flasks with bacterium and bionanocomposite. These four flasks were incubated at 37 °C for 2 h. Next, 0.1 mℓ of the content of each flask was added in Mueller Hinton agar, and the dishes were kept at 37 °C for 24 h. The antibacterial performance of bionanocomposite was investigated by counting the colonies on agar plates after 24 h.

#### Antibacterial activity screening (OD study)

First, four flasks with 10 ml Nutrient Broth were prepared. In the following two tube tests with *Escherichia coli, Staphylococcus aureus* plus 10 ml sterile physiological serum with 0.5 McFarland turbidity standard was adjusted. Then about 1 ml from the suspensions of the bacterium in each tube test was added to the four flasks. Two of these flasks have just bacterium and two of them have bacterium and bionanocomposite (0.01 g). In a typical experiment for the construction of the growth curves, all bacterial cultures with an approximate concentration of 10^6^–10^7^ colony-forming units per milliliter (CFU/mL) were inoculated into a Nutrient Broth medium. To ensure optimum contact between chitosan-AgIO_3_ bionanocomposite and bacterial cells, all experiments were performed in an incubator shaker at 37 °C and 180 rpm. The rate of bacterial killing was checked at various time intervals in terms of the UV–visible spectra. Two cultures of the bionanocomposite-free medium under the same growth bacteria conditions were used as controls. To avoid potential optical interference during optical measurements of the growing cultures caused by the light-scattering properties of the nanoparticles, the same liquid medium without microorganisms, but containing the same concentration of bionanocomposite cultured under the same conditions as blank controls.

## Result and discussion

One of the most critical and practical approaches in organic chemistry is the design and synthesis of nanocomposites with natural polymers derived from renewable resources and the investigation of their remarkable performance in medical fields. Among various areas in drug discovery design, synthesis and introduce novel antibacterial agents is extremely important. In this research, chitosan-AgIO_3_ bionanocomposite was synthesized through an easy method with low cost and readily available materials. Briefly, upon the addition of AgNO_3_, the amino and hydroxyl groups on the structure of chitosan coordinated Ag^+^ ions tightly to form a suspension of chitosan-Ag^+^. In the following with adding KIO_3_, Ag ions reacted to KIO_3_ to generate AgIO_3_ nanoparticles. Eventually, chitosan-AgIO_3_ bionanocomposite was applied for antibacterial features against several bacteria. The structural corroboration of bionanocomposite is studied by utilizing analytical techniques including FT-IR spectra for detecting related functional groups, SEM for determination of morphology and structure, EDX analysis for elemental confirmation, and XRD pattern for study the crystal structure of bionanocomposite.

### Investigation of chitosan-AgIO_3_ bionanocomposite characteristics

#### Fourier transform infrared spectroscopy of chitosan-AgIO_3_ bionanocomposite

**Figure 2:**
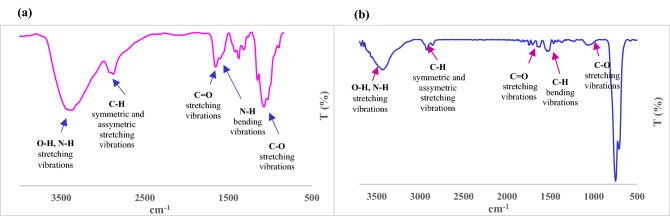
FT-IR spectra of (**a**) chitosan, and (**b**) chitosan-AgIO_3_ bionanocomposite.

According to Fig. 2, the fabrication of chitosan-AgIO_3_ was determined by the FT-IR spectroscopy technique. As can be seen in Fig. 2a, the characteristic absorption bands for chitosan were shown at 3413 cm^−1^ as a strong peak for stretching vibrations for both O–H and N–H, overlapped, 2918 cm^−1^ is for C–H symmetric stretching vibrations, 2873 cm^−1^ is for C–H asymmetric stretching vibrations. The bands confirmed the presence of residual N-acetyl groups at around 1654 cm^−1^ for C=O stretching of amide and 1593 cm^−1^ for N–H bending vibrations. The peak at 1518 cm^−1^ is illustrated C–H bending vibrations and 1375 cm^−1^ is represented CH_3_ symmetrical deformation. The band at 1315 cm^−1^ is indicated the C–N stretching vibration of amide, and 1259 cm^−1^ is related to the bending vibration of hydroxyl groups present in chitosan. The asymmetric stretching of the C–O–C bridge is determined by an absorption peak at 1153 cm^−1^. The bands at 1072 and 1029 cm^−1^ correspond to the C–O stretching vibrations. Compared to the FT-IR spectrum of chitosan, the FT-IR spectrum of bionanocomposite demonstrates variation (Fig. 2b). The presence of absorption peaks at 710 cm^−1^ and 748 cm^−1^ correspond to AgIO_3_ nanoparticles which represent the presence of AgIO_3_ in the final bionanocomposite. On the basis of the data find from these two spectra, a reason for the shifting of wavenumbers and lower intensities of peaks is confirming interactions between Ag, O, and N atoms. In addition, C=O stretching band at 1654 cm^−1^ and N–H absorption band at 1593 cm^−1^ shifted to lower frequency owing to the bind between chitosan and AgIO_3_.

#### Energy dispersive X-ray spectroscopy of chitosan-AgIO_3_ bionanocomposite

**Figure 3:**
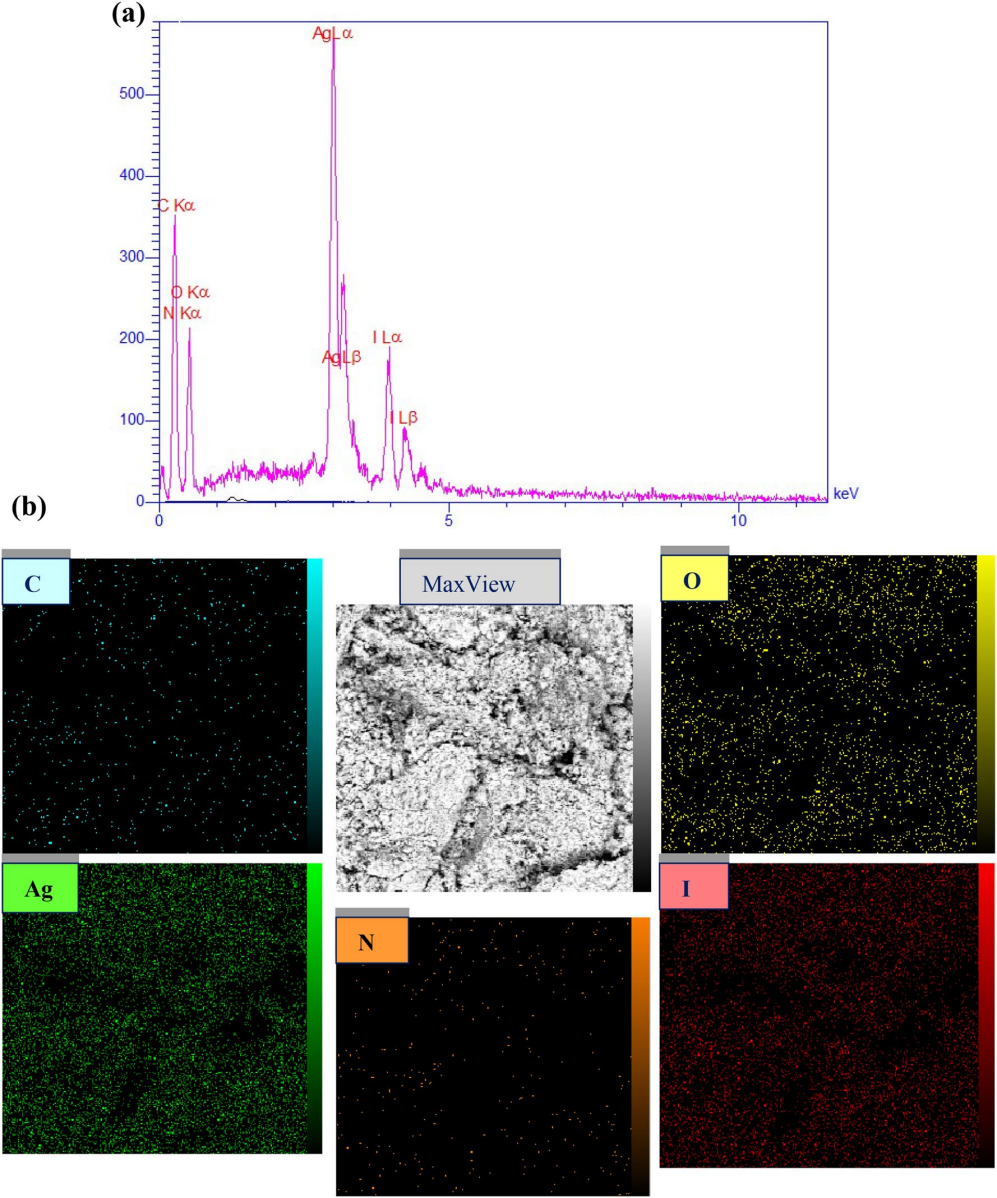
(**a**) EDX analysis of chitosan-AgIO_3_ bionanocomposite, and (**b**) Elemental mapping of chitosan-AgIO_3_ bionanocomposite.

EDX analysis was performed to study the presence of elements in the structure of the bionanocomposite (Fig. 3a). EDS spectrum further represented C, O, N, Ag, and I elements in prepared bionanocomposite, and the atomic ratio between Ag and I was about 1:1, representing that chitosan-AgIO_3_ bionanocomposite was successfully synthesized. In addition, the elemental mapping of EDX patterns shows the presence of C, O, N, Ag, and I elements in the bionanocomposite (Fig. 3b).

#### Scanning electron microscopy of chitosan-AgIO_3_ bionanocomposite

**Figure 4:**
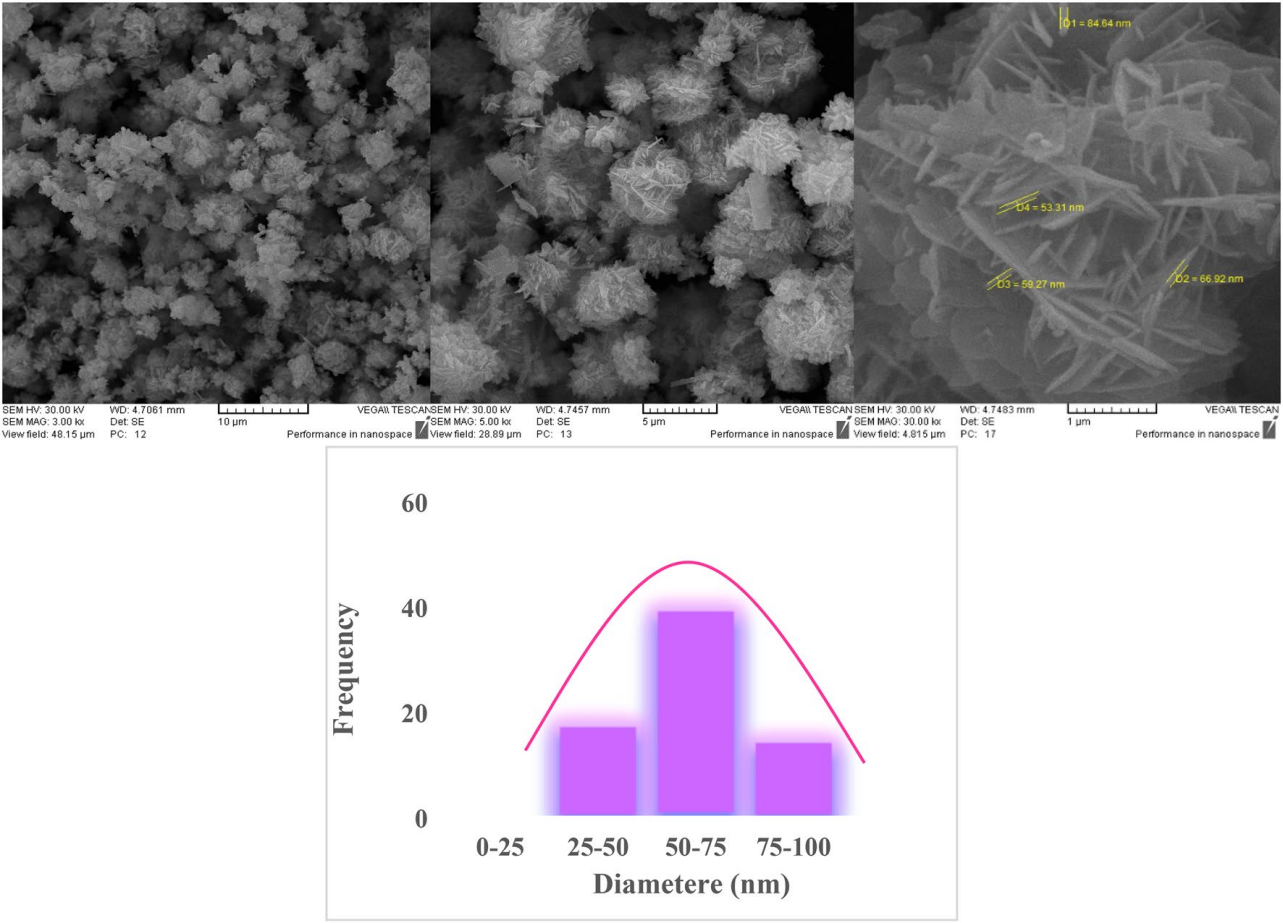
SEM images of chitosan-AgIO_3_ bionanocomposite and the particle size distribution diagram of the chitosan-AgIO_3_ bionanocomposite.

To study the morphology and structure of chitosan-AgIO_3_ bionanocomposite, the SEM analysis was done (Fig. 4). As seen in the figure, it is clear that AgIO_3_ nanoparticles have covered the structure of chitosan. The presence and uniform distribution of AgIO_3_ nanoparticles on the surface of chitosan is well observed. According to the SEM image, the average size of synthesizing AgIO_3_ nanoparticles is less than 60 nm. To specify the size of nanoparticles, 70 particles were selected randomly. The average particle size of nanoparticles, is about 57 nm.

#### X-ray diffraction analysis of chitosan-AgIO_3_ bionanocomposite

**Figure 5:**
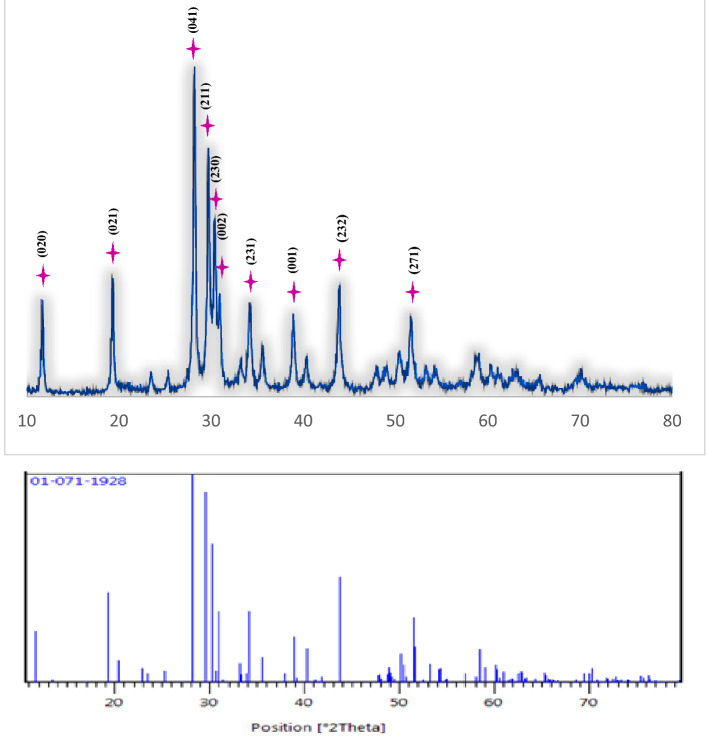
The XRD pattern of chitosan-AgIO_3_ bionanocomposite.

To investigate the structure of the inorganic nanoparticles to verify the formation of AgIO_3_ nanoparticles in the bionanocomposite, the XRD pattern was prepared. As shown in Fig. 5, the bionanocomposite exhibited the main peaks, consistent with the characteristic peaks of AgIO_3_ (JCPDS 01-071-1928). The diffraction peaks of AgIO_3_ at 2θ values of 11.75°, 19.37°, 28.21°, 29.73°, 30.41°, 30.95°, 34.23°, 38.94°, 43.90°, and 51.69° were assigned to (020), (021), (041), (211), (230), (002), (231), (001), (232) and (271) crystal planes of the orthorhombic AgIO_3_, respectively^44^. Besides, the size of the nanoparticles characterized by X-ray line broadening using the Scherrer equation (D = kλ/β cos θ) was about 29 nm.

### Study the antibacterial activity of the chitosan-AgIO_3_ bionanocomposite

#### Zone of inhibition studies

**Figure 6:**
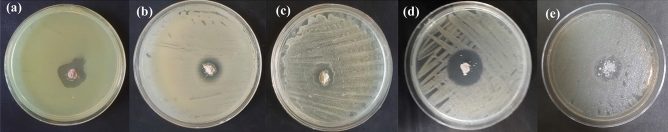
Inhibition zones of chitosan-AgIO_3_ against (**a**) *Pseudomonas aeruginosa;* (**b**) *Klebsiella pneumoniae*; (**c**) *Staphylococcus saprophyticus*; (**d**) *Escherichia coli*; (**e**) *Staphylococcus aureus* bacteria for 24 h.

**Figure 7:**
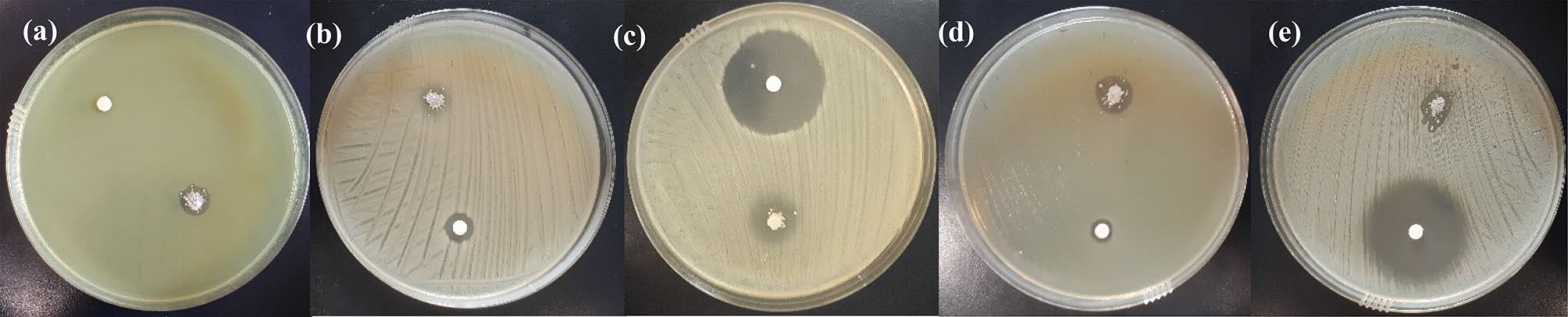
Inhibition zones of chitosan-AgIO_3_ against (**a**) *Pseudomonas aeruginosa*; (**b**) *Klebsiella pneumoniae*; (**c**) *Staphylococcus saprophyticus*; (**d**) *Escherichia coli*; (**e**) *Staphylococcus aureus* bacteria for 24 h compared with 0.001 g/ml penicillin.

**Figure 8:**
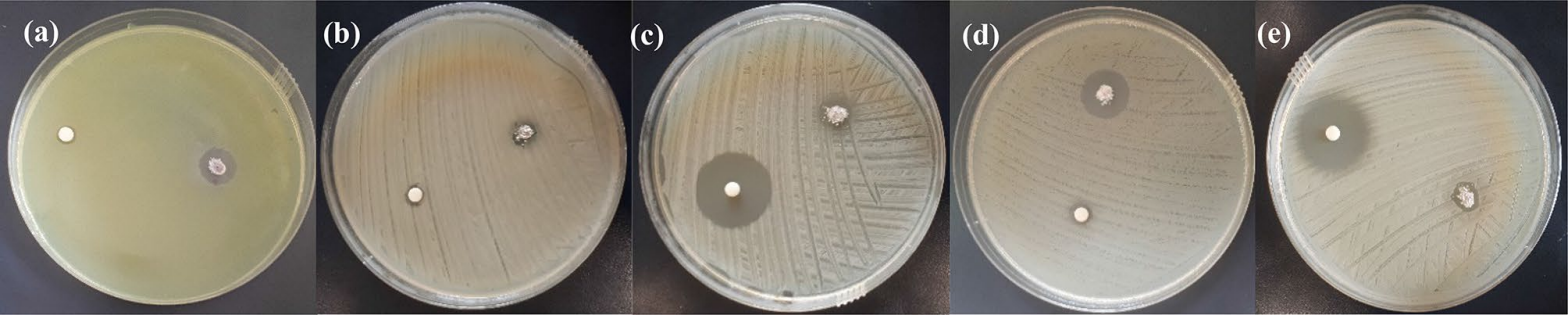
Inhibition zones of chitosan-AgIO_3_ against (**a**) *Pseudomonas aeruginosa;* (**b**) *Klebsiella pneumoniae*; (**c**) *Staphylococcus saprophyticus*; (**d**) *Escherichia coli;* (**e**) *Staphylococcus aureus* bacteria for 24 h compared with 0.001 g/ml silver sulfadiazine.

The evaluation of the antibacterial performance of the synthesized chitosan-AgIO_3_ bionanocomposite against *Pseudomonas aeruginosa, Klebsiella pneumoniae, Staphylococcus saprophyticus, Escherichia coli, Staphylococcus aureus* by in vitro study and agar diffusion method was done. Investigation of the inhibition zone in millimeters (mm) around the bionanocomposite (0.01 g) was measured to determine its antibacterial efficacy against these five types of microorganisms. The growth inhibition zone of bionanocomposite against all five bacteria was shown in Fig. 6. In addition, the details of the inhibition zone width of each bacteria are listed in Table 1. These results properly demonstrate the high performance of chitosan-AgIO_3_ for killing various bacteria. In order to evaluate and expand the performance of the bionanocomposite against these five bacteria, the efficacy of the bionanocomposite was studied in comparison with penicillin and silver sulfadiazine as commercial antibiotics with different concentrations of the penicillin and silver sulfadiazine and constant amount of bionanocomposite (0.01 g). The inhibition zone in millimeters (mm) around the bionanocomposite (0.01 g) and penicillin (0.001 g/ml) (Fig. 7) and silver sulfadiazine (0.001 g/ml) (Fig. 8) were summarized (see Tables S1 and S2 in Supplementary Information). Inhibition zone measurements around the bionanocomposite (0.01 g) and penicillin (0.01 g/ml) and silver sulfadiazine (0.01 g/ml) were further conducted (see Figs. S1 and S2 and Tables S3 and S4 in Supplementary Information) to determine the influence of concentration in antibacterial activity. These findings represent that chitosan-AgIO_3_ has a high potential for eradication of various microorganisms. Moreover, the antibacterial activity of the chitosan-AgIO_3_ bionanocomposite was compared with the relevant antibacterial materials reported in the literature. Data about the ZOI (mm) toward the target bacteria were summarized in Table 2.

**Table 1:** Width of inhibition zones for chitosan-AgIO_3_ bionanocomposite against (a) *Pseudomonas aeruginosa*; (b) *Klebsiella pneumoniae;* (c) *Staphylococcus saprophyticus;* (d) *Escherichia coli;* (e) *Staphylococcus aureus* bacteria.

Bacteria	Inhibition zone (diameter), mm
*Pseudomonas aeruginosa*	8
*Klebsiella pneumoniae*	6
*Staphylococcus saprophyticus*	4
*Escherichia coli*	8
*Staphylococcus aureus*	6

**Table 2:** Example reports on the antibacterial activity of Ag-based materials.

Sample	Bateria	ZOI (mm)	References
Fe_3_O_4_@Cs@Ag	*S. aureus*	8	^45^
	*B. subtilis*	6.33	
	*E. faecalis*	8.66	
	*E. coli*	9	
	*P. mirabilis*	8.33	
	*P. aeruginosa*	7.66	
Silver nanoparticle (triangle shaped)	*P. aeruginosa*	3	^46^
	*E. coli*	1.4	
Silver nanoparticle (spherical)	*P. aeruginosa*	8	
	*E. coli*	1.5	
Fe_3_O_4_/PVA-10%Ag	*E. coli*	2.22	^47^
	*S. aureus*	3.09	
Ag/Fe_3_O_4_@SiO2-IT	*E. coli*	2.13	^48^
	*S. aureus*	2.98	
Ag/ZnO-clay	*E. coli*	6	^49^
	*E. faecalis*	10	
CNT-Ag (carbon nanotube)	*E. coli*	3.5	^50^
GO-Ag (graphene oxide)	*E. coli*	2.5	
Chitosan-AgIO_3_	*P. aeruginosa*	8	This work
	*Klebsiella pneumoniae*	6	
	*Staphylococcus sp.*	4	
	*E. coli*	8	
	*S. aureus*	6	

#### Plate-count method

**Figure 9:**
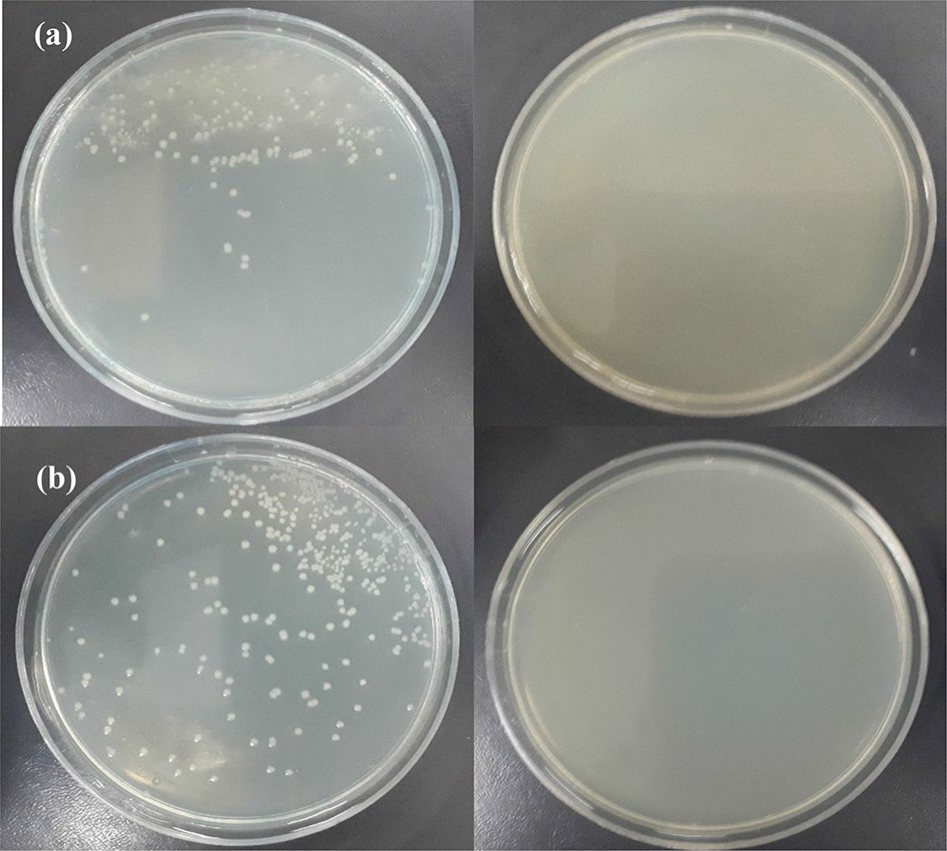
Images of (**a**) *Staphylococcus aureus* and (**b**) *Escherichia coli* in the absence and presence of bionanocomposite after 24 h.

The colony plate images of *Staphylococcus aureus* (ATCC 12600) and *Escherichia coli* (ATCC 9637) bacteria in the presence of chitosan-AgIO_3_ bionanocomposite (0.01 g) are represented in Fig. 9. As illustrated in the figure, all colonies of *Staphylococcus aureus* and *Escherichia coli* were killed by treatment with the chitosan-AgIO_3_ bionanocomposite.

#### Optical density

**Figure 10:**
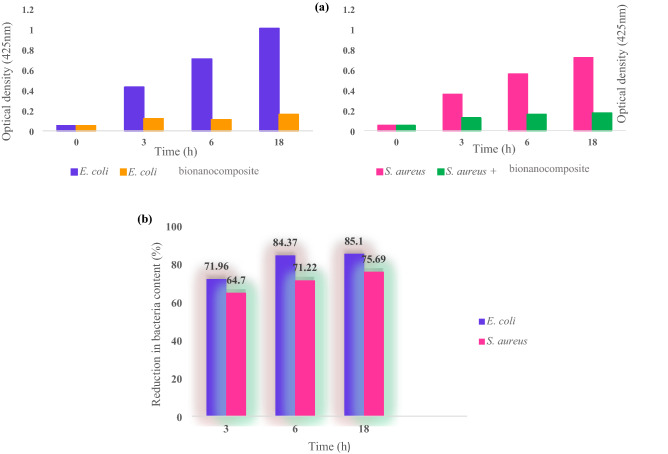
(**a**) The effect of the chitosan-AgIO_3_ bionanocomposite on the inhibition of bacterial growth for *Escherichia coli* and *Staphylococcus aureus and* (**b**) the percentage of reduction in bacteria content after 3, 6, and 18 h by OD meseaurments.

The OD measurements of bacterial cultures were investigated in the presence of 0.01 g chitosan-AgIO_3_ bionanocomposite, 0.5 McFarland turbidity standard, and Nutrient Broth media. Growing cultures were checked at various times, including 3, 6, and 18 h (Fig. 10). As can be seen in a bar diagram in Fig. 10a, the antibacterial property of bionanocomposite demonstrate a considerable inhibition of bacterial growth for *Escherichia coli* and *Staphylococcus aureus.* As represented in a chart in Fig. 10b, for *Escherichia coli* after 3 h, 71.96% of bacteria content decreased with the presence of bionanocomposite, and this growth inhibition was 84.37% after 6 h. After 18 h, the reduction in bacteria content is 85.1%. For *Staphylococcus aureus* after 3 h, 64.7% of bacteria content decreased in the presence of bionanocomposite. After 6 h, this reduction in bacteria content was about 71.22%, and at least after 18 h, 75.69% of bacteria content was reduced. Based on the present findings of this research, the chitosan-AgIO_3_ bionanocomposite introduces as an antibacterial agent for killing and inhibiting bacterial growth in terms of the UV absorption spectra.

### Mechanism for antibacterial activity of bionanocomposite

**Figure 11:**
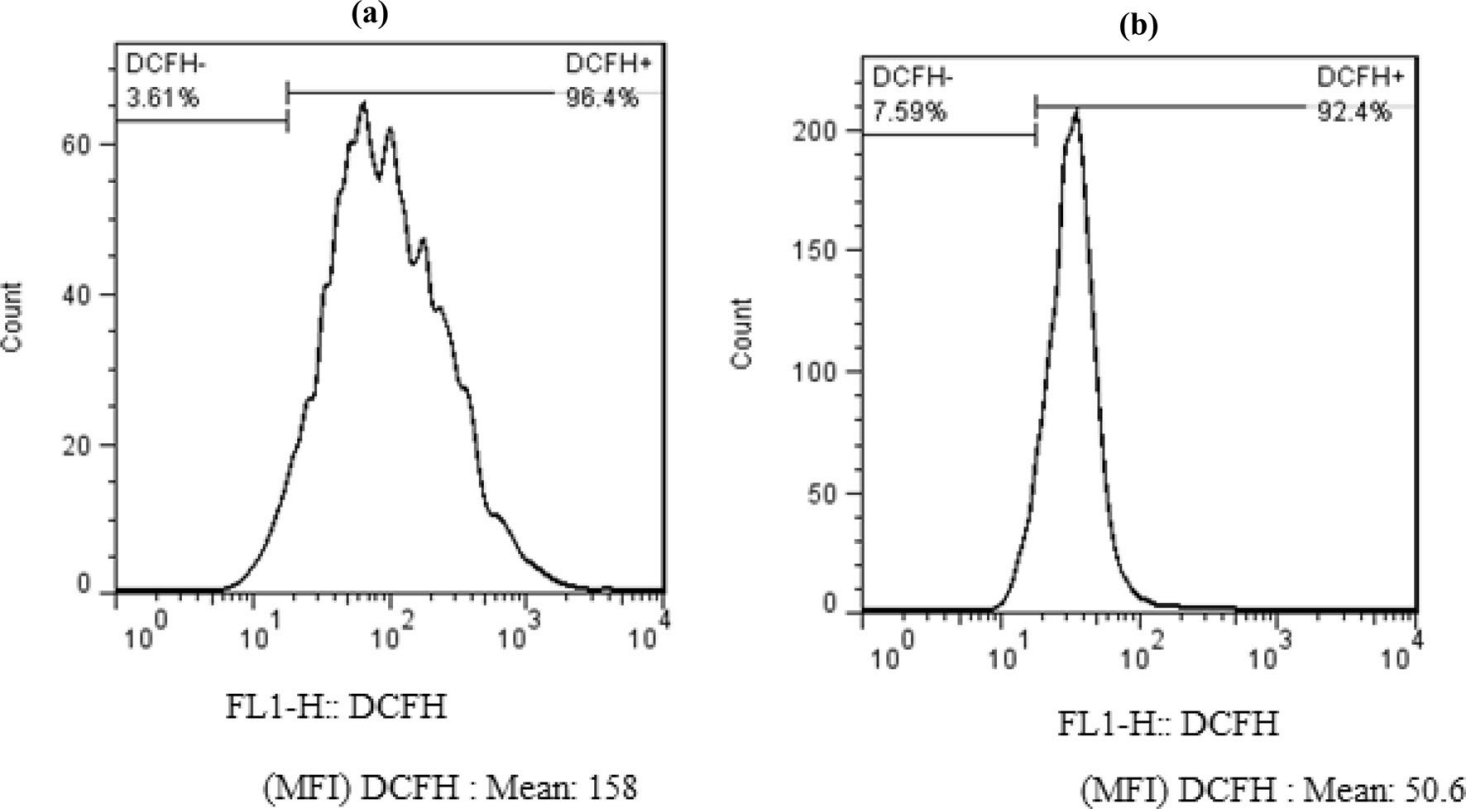
The flow cytometry analysis of (**a**) bionanocomposite and, (**b**) control group with *Escherichia coli.*

**Figure 12:**
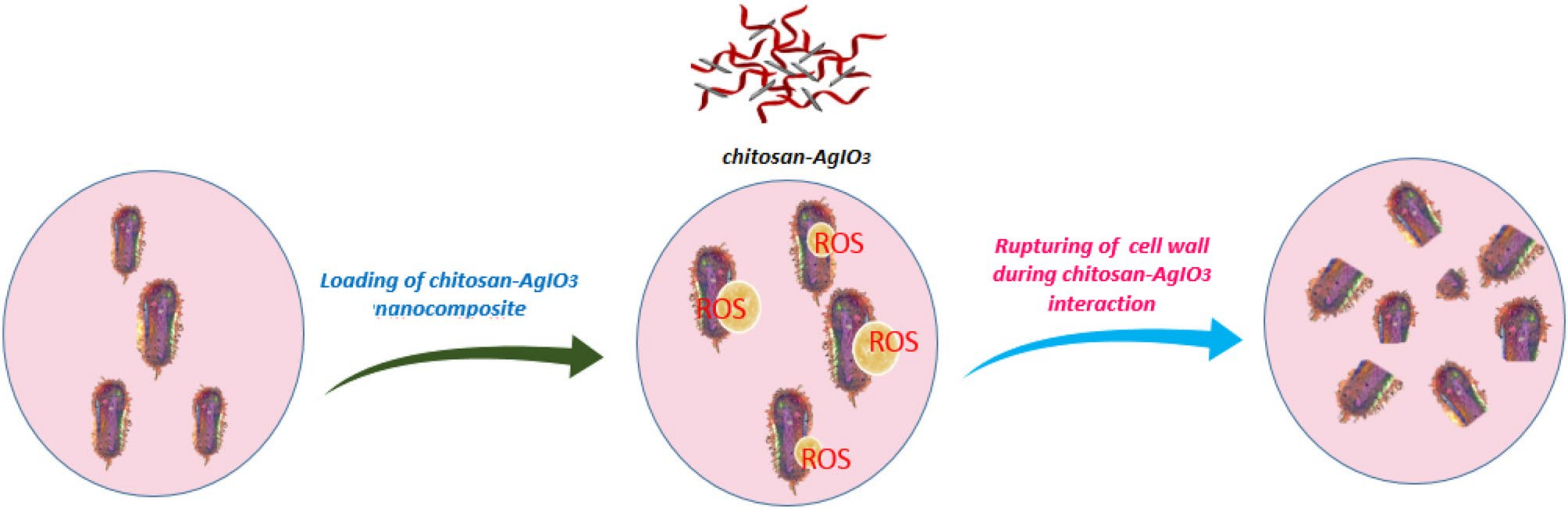
Possible antibacterial mechanism of chitosan-AgIO_3_ bionanocomposite.

The mechanism of the antimicrobial efficacy of chitosan-AgIO_3_ bionanocomposite was further studied on *Escherichia coli* by flow cytometry. It is reputable that ROS is a fundamental factor in the antibacterial activities of nanomaterials, which could directly damage the cell membrane, phospholipids, and/or membrane proteins. Reactive oxygen species as free radicals cause damage to a variety of pathogens^51,52^. Nanomaterials with ROS-producing abilities could be a beneficial strategy to fight against bacteria. To explore the antibacterial mechanism of bionanocomposite, the production of ROS was measured by DCFDA, a commercial fluorescent probe. As can be seen in Fig. 11, the fluorescence intensity in the diagram of bionanocomposite increased, and the stronger the fluorescence intensity, the greater the number of dead cells which means a good antibacterial activity of bionanocomposite. The fluorescence intensity of bionanocomposite was stronger than the control group and the MFI parameter of bionanocomposite is three times more than the control group which means the dead cells in bionanocomposite are highly increased. As a consequence, the bacterium was damaged due to the membrane damage caused by ROS production, thereby achieving antibacterial activity (Fig. 12).

## Conclusion

Due to the increase in microbial resistance to existing antimicrobial agents, which unfortunately increases the percentage of disease and mortality, the introduction and synthesis of new antimicrobial agents are essential. To introduce new antimicrobial agents in this study, chitosan-AgIO_3_ nanocomposite was successfully designed and synthesized for the first time with a simple method and with utilizing readily available materials. Chitosan is a natural polymer and was used as the base of the bionanocomposite and AgIO_3_ nanoparticles were immobilized on the structure of chitosan. The presence of chitosan in the bionanocomposite cause biodegradable and environmentally friendly nanocomposite. The structural features of bionanocomposite were studied with several techniques including FT-IR, EDX, SEM, and XRD analyses, and the results verified the effective immobilization of AgIO_3_ on chitosan. The antibacterial efficacy was evaluated through the agar diffusion strategy, plate count method, and optical density study with different microorganisms. Besides the antibacterial efficacy of the bionanocomposite was compared with two commercial drugs and in some cases, the synthesized bionanocomposite has better performance against bacteria in comparison with common drugs. In a view of the difficult eradication of *Pseudomonas aeruginosa* due to its considerable capacity to resist antibiotics, presenting a new antibacterial agent which has a good operation against this bacteria is very important. In addition, chitosan-AgIO_3_ bionanocomposite as an environment-friendly and efficient antibacterial agent is highly recommended in a water purification process, and biomedical applications due to its specific properties such as high antibacterial activity, low-cost, simple, and green synthesis method. The findings represented that this work could open a way for producing a novel low-cost, biodegradable bionanocomposite with high performance against different bacteria. Due to the effectiveness of the synthesized bionanocomposite compared with antibiotics, it could be introduced as a good suggestion in therapeutic applications as well as burn wounds. The main impediment of this work is the separation of the bionanocomposite which is designed in our future research as well as other applications of chitosan-AgIO_3_ bionanocomposite.

## Supplementary Information

Supplementary Information.

## Data Availability

Additional supplementary information includes the table of the width of the inhibition zone in millimeters (mm) around the bionanocomposite (0.01 g) against various bacteria and compared it with 0.001 g/ml penicillin and silver sulfadiazine, besides these tables for 0.01 g/ml penicillin and silver sulfadiazine. In addition, figures related to the inhibition zones of chitosan-AgIO_3_ against various bacteria for 24 h compared with 0.01 g/ml penicillin and silver sulfadiazine can be found in the online version of this article on the publisher’s website.
