# Supplementary material for: Design, synthesis, and characterization of novel eco-friendly chitosan-AgIO3 bionanocomposite and study its antibacterial activity
Source: Sci Rep. 2022 Jun 21;12:10491. doi: 10.1038/s41598-022-14501-6 (PMC9213402; doi:10.1038/s41598-022-14501-6)
Supplement: Supplementary file 1 — Supplementary Information. [file 41598_2022_14501_MOESM1_ESM.pdf]

Design, synthesis, and characterization of novel eco-friendly chitosan-$AgIO_3$ bionanocomposite and study its antibacterial activity

*Mohammad Ali Ahghari, Mohammad Reza Ahghari, Maryam Kamalzare and Ali Maleki**

Catalysts and Organic Synthesis Research Laboratory, Department of Chemistry, Iran University of Science and Technology, Tehran 16846-13114, Iran

E-mail: maleki@iust.ac.ir; Fax: +98 21 73021584; Tel: +98 21 73228313

Table of contents

Subject	Page

Table S1. Width of inhibition zones for chitosan-$AgIO_3$ bionanocomposite against a) *Pseudomonas aeruginosa*; b) *Klebsiella pneumoniae;* c) *Staphylococcus saprophyticus;* d) *Escherichia coli; e*) *Staphylococcus aureus* bacteria compared with 0.001 g/ml penicillin for 24 h …………..…………………………………………..…..….……S3

Table S2. Width of inhibition zones for chitosan-$AgIO_3$ bionanocomposite against a) *Pseudomonas aeruginosa*; b) *Klebsiella pneumoniae;* c) *Staphylococcus saprophyticus;* d) *Escherichia coli; e*) *Staphylococcus aureus* bacteria compared with 0.001 g/ml silver sulfadiazine for 24 h ……..……………………………………….………...…S4

Table S3. Width of inhibition zones for chitosan-$AgIO_3$ bionanocomposite against a) *Pseudomonas aeruginosa*; b) *Klebsiella pneumoniae;* c) *Staphylococcus saprophyticus;* d) *Escherichia coli; e*) *Staphylococcus aureus* bacteria compared with 0.01 g/ ml penicillin for 24 h…..………...………………………………………...…………S5

Table S4. Width of inhibition zones for chitosan-$AgIO_3$ bionanocomposite against a) *Pseudomonas aeruginosa*; b) *Klebsiella pneumoniae;* c) *Escherichia coli* bacteria compared with 0.01 g/ ml silver sulfadiazine for 24 h ……….S6

Figure S1. Inhibition zones of chitosan-$AgIO_3$ against a) *Pseudomonas aeruginosa;* b) *Klebsiella pneumoniae;* c) *Staphylococcus saprophyticus***;** d) *Escherichia coli;* e) *Staphylococcus aureus* bacteria for 24 h compared with 0.01 g/ml penicillin ……………………………………….…………………………………….…………S7

Figure S2. Inhibition zones of chitosan-$AgIO_3$ against a) *Pseudomonas aeruginosa;* b) *Klebsiella pneumoniae;* c) *Escherichia coli* bacteria for 24 h compared with 0.01 g/ml silver sulfadiazine ……..…S8

Table S1. Width of inhibition zones for chitosan-$AgIO_3$ bionanocomposite against a) *Pseudomonas aeruginosa*; b) *Klebsiella pneumoniae;* c) *Staphylococcus saprophyticus;* d) *Escherichia coli; e*) *Staphylococcus aureus* bacteria compared with 0.001 g/ml penicillin for 24 h

**Bacteria**	**Inhibition zone (diameter), mm**<br>**Bionanocomposite (0.01 g)**	**Inhibition zone (diameter), mm**<br>**Penicillin (0.001 g/ml)**
Pseudomonas aeruginosa	4	0.5
Klebsiella pneumoniae	2	5
Staphylococcus saprophyticus	10	19
Escherichia coli	7	2
Staphylococcus aureus	7	17

Table S2. Width of inhibition zones for chitosan-$AgIO_3$ bionanocomposite against a) *Pseudomonas aeruginosa*; b) *Klebsiella pneumoniae;* c) *Staphylococcus saprophyticus;* d) *Escherichia coli; e*) *Staphylococcus aureus* bacteria compared with 0.001 g/ml silver sulfadiazine for 24 h

**Bacteria**	**Inhibition zone (diameter), mm Bionanocomposite (0.01 g)**	**Inhibition zone (diameter), mm Silver sulfadiazine (0.001 g/ml)**
Pseudomonas aeruginosa	5	2
Klebsiella pneumoniae	2	1.5
Staphylococcus saprophyticus	5	13
Escherichia coli	7	3
Staphylococcus aureus	5.5	14

Table S3. Width of inhibition zones for chitosan-$AgIO_3$ bionanocomposite against a) *Pseudomonas aeruginosa*; b) *Klebsiella pneumoniae;* c) *Staphylococcus saprophyticus;* d) *Escherichia coli; e*) *Staphylococcus aureus* bacteria compared with 0.01 g/ml penicillin for 24 h

Bacteria	Inhibition zone (diameter), mm Bionanocomposite (0.01 g)	Inhibition zone (diameter), mm penicillin (0.01 g/ml)
Pseudomonas aeruginosa	4	1.5
Klebsiella pneumoniae	3	8
Staphylococcus saprophyticus	6	21
Escherichia coli	6	8
Staphylococcus aureus	6	22

Table S4. Width of inhibition zones for chitosan-$AgIO_3$ bionanocomposite against a) *Pseudomonas aeruginosa*; b) *Klebsiella pneumoniae;* c) *Escherichia coli* bacteria compared with 0.01 g/ml silver sulfadiazine for 24 h

**Bacteria**	**Inhibition zone (diameter), mm**<br>**Bionanocomposite (0.01 g)**	**Inhibition zone (diameter), mm**<br>**Silver sulfadiazine (0.01 g/ml)**
Pseudomonas aeruginosa	4	12
Klebsiella pneumoniae	2	2
Escherichia coli	5	13

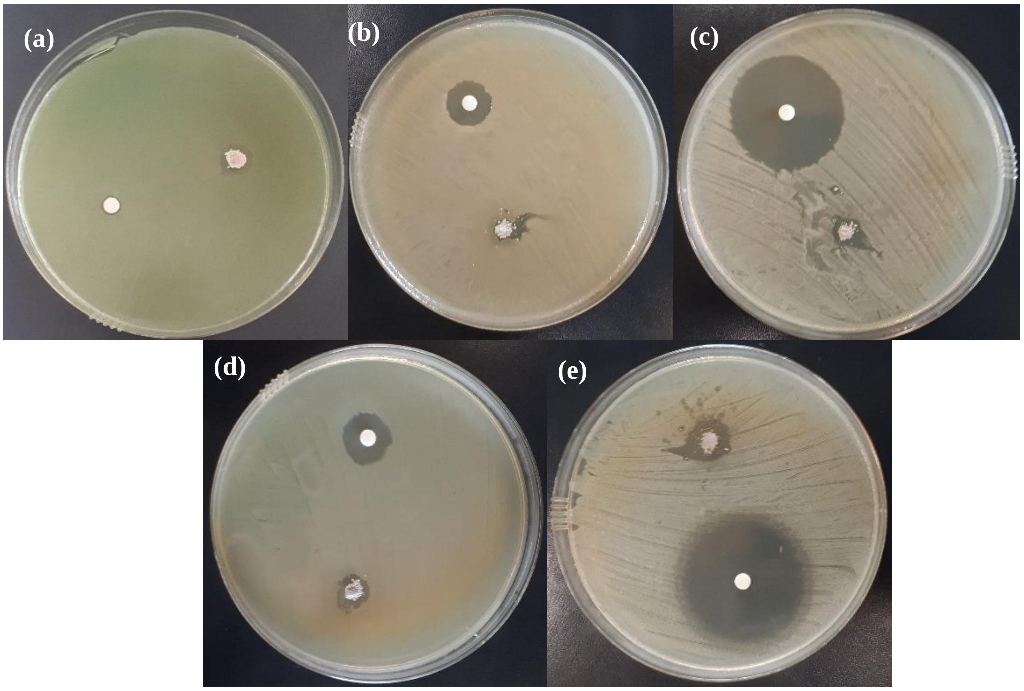

Figure S1. Inhibition zones of chitosan-$AgIO_3$ against a) *Pseudomonas aeruginosa;* b) *Klebsiella pneumoniae;* c) *Staphylococcus saprophyticus***;** d) *Escherichia coli;* e) *Staphylococcus aureus* bacteria for 24 h compared with 0.01 g/ml penicillin

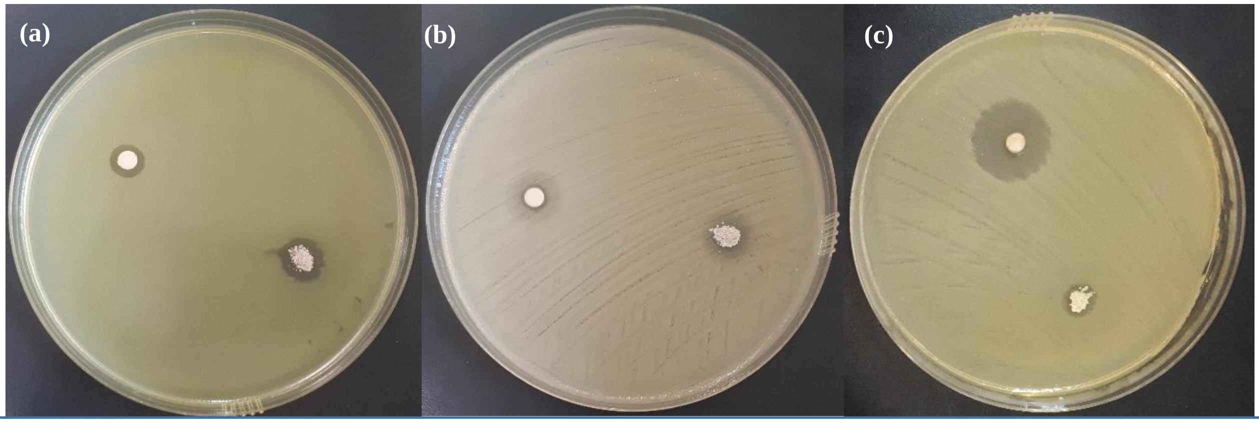

Figure S2. Inhibition zones of chitosan-$AgIO_3$ against a) *Pseudomonas aeruginosa;* b) *Klebsiella pneumoniae;* c) *Escherichia coli* bacteria for 24 h compared with 0.01 g/ml silver sulfadiazine
